# Cardiovascular events and death after catheter ablation in very old patients with nonvalvular atrial fibrillation

**DOI:** 10.18632/aging.204952

**Published:** 2023-08-14

**Authors:** Keisuke Okawa, Satoshi Taya, Takeshi Morimoto, Ryu Tsushima, Yuya Sudo, Ai Sakamoto, Eisuke Saito, Masahiro Sogo, Masatomo Ozaki, Masahiko Takahashi

**Affiliations:** 1 Department of Cardiovascular Medicine, Kagawa Prefectural Central Hospital, Takamatsu, Kagawa 760-8557, Japan; 2 Department of Clinical Epidemiology, Hyogo Medical University, Nishinomiya, Hyogo 663-8501, Japan

**Keywords:** atrial fibrillation, catheter ablation, cardiovascular event, cardiovascular death, very old patient

## Abstract

Background: Catheter ablation of atrial fibrillation (AF) is recommended for selected older patients. However, the preventive effects of AF ablation on cardiovascular events and death remain unclear, especially in older patients. This study aimed to investigate the impact of AF ablation on the incidence of cardiovascular events and death in very old nonvalvular AF (NVAF) patients.

Methods: We conducted a prospective cohort study of consecutive patients with NVAF aged ≥80 years and using direct oral anticoagulants (DOACs). We defined cardiovascular events as acute heart failure (AHF), strokes and systemic embolisms (SSEs), acute coronary syndrome (ACS), and sudden cardiac death (SCD) and cardiovascular death as AHF/SSE/ACS-related death and SCD. We compared the 3-year incidence of cardiovascular events and death between the patients who underwent AF ablation (Ablation group) and those who received medical therapy only (Medication group).

Results: Among the 782 NVAF patients using DOACs, propensity score matching provided 208 patients in each group. The Ablation group had a significantly lower 3-year incidence of cardiovascular events and death than the Medication group: cardiovascular events, 24 (13.2%) vs. 43 (23.3%), log-rank *P* = 0.009 and hazard ratio (HR) 0.52 (95% confidence interval (CI) 0.32–0.86) and cardiovascular deaths, 5 (3.0%) vs. 15 (7.8%), log-rank *P* = 0.019 and HR 0.32 (95% CI 0.16–0.88).

Conclusions: In very old NVAF patients using DOACs, those who underwent AF ablation had a lower incidence of both cardiovascular events and death than those who received medical therapy only.

## INTRODUCTION

Atrial fibrillation (AF) is associated with cardiovascular events such as strokes and heart failure [1]. An appropriate therapy in older patients with AF is required because the incidence of both AF and fatal AF-related cardiovascular events increases with age [2, 3]. Several studies have shown that catheter ablation of AF is associated with a lower incidence of ischemic strokes, heart failure, and mortality [4, 5]. However, the effects of AF ablation on the prognosis of patients remain controversial, especially among older people [6]. In fact, most clinicians prefer to give older AF patients rate control strategy medications in the real-world practice [7]. Furthermore, direct oral anticoagulant (DOAC) use can prevent ischemic strokes and is safe even in very old AF patients [8–10].

A recent international consensus statement has classified AF ablation for selected older patients as a class IIa recommendation based on studies demonstrating its acceptable procedural efficacy and safety [11]. However, few studies have evaluated the impact of AF ablation on the long-term cardiovascular prognosis in older patients. Thus, we investigated the 3-year outcomes in AF patients aged ≥80 years and using DOACs. In addition, we compared the incidence of cardiovascular events and death between patients who underwent AF ablation and those who received medical therapy only.

## RESULTS

### Baseline characteristics

**Figure 1:**
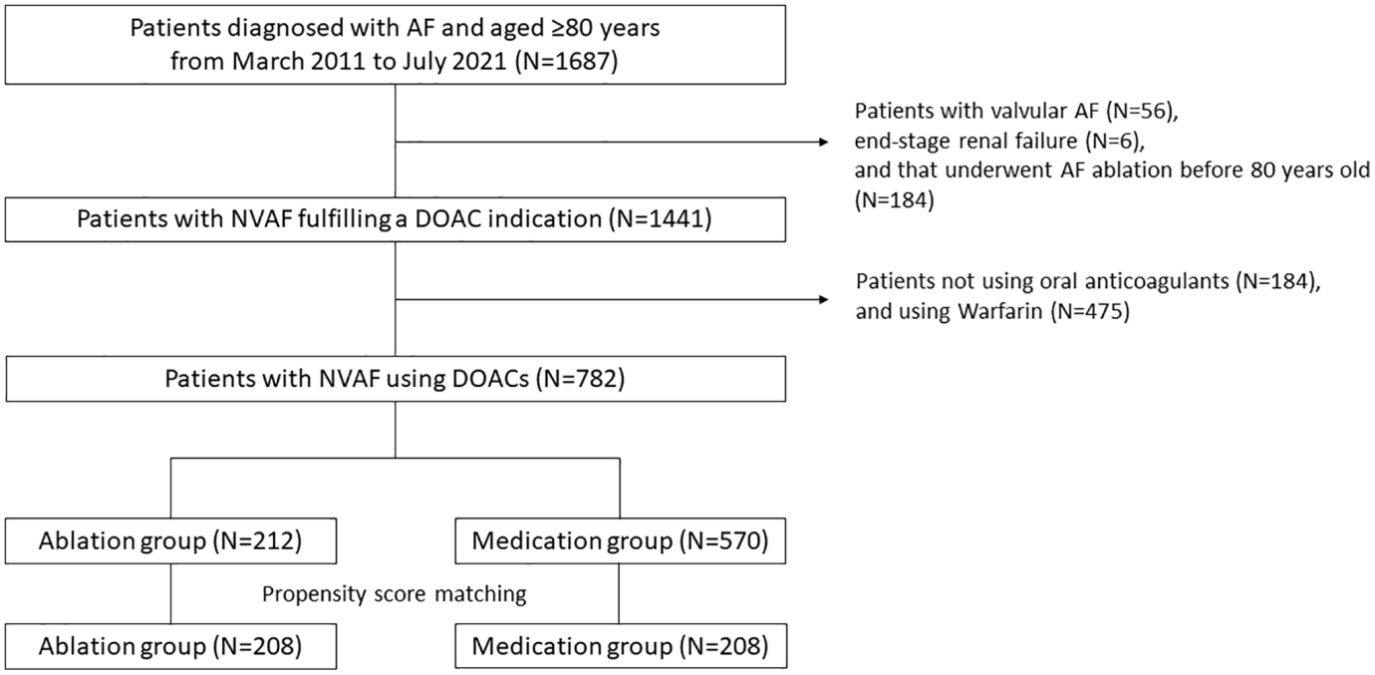
**Flowchart of the study procedure.** A total of 1,687 patients aged ≥80 years were diagnosed with AF. Ultimately, 782 patients with NVAF using DOACs (Ablation group, *n* = 212; Medication group, *n* = 570) were included in this study as a crude cohort. After propensity score matching, 208 patients were extracted from each group. Abbreviations: AF: atrial fibrillation; NVAF: nonvalvular AF; DOAC: direct oral anticoagulant.

Of the 1,687 patients aged ≥80 years diagnosed with AF, 56 with valvular AF, 6 with end-stage renal failure, 184 who underwent AF ablation before 80 years old, 184 not using DOACs, and 475 using warfarin were excluded. Among the 782 patients with Nonvalvular AF (NVAF) using DOACs, 212 underwent catheter ablation of AF (Ablation group), and 570 received medical therapy only (Medication group) (Figure 1).

In the original total cohort, the median follow-up period was 35 and 36 months in the Ablation and Medication groups, respectively. The mean age, prevalence of hypertension and heart failure, proportion of patients with a history of thromboembolisms, and a CHADS_2_ score were significantly lower in the Ablation group than in the Medication group. The creatinine clearance values were significantly higher in the Ablation group than in the Medication group. In the Medication group, a rhythm control strategy using antiarrhythmic drugs was adopted in 57 (10%) patients. Regarding the transthoracic echocardiogram (TTE) parameters, the patients in the Ablation group had a significantly lower left atrium (LA) diameter and higher left ventricular ejection fraction (LVEF) than those in the Medication group (Table 1).

**Table 1:** Baseline characteristics (original total cohort).

	**Ablation group (*N* = 212)**	**Medication group (*N* = 570)**	***P*-value**
Follow-up period, months, median (IQR)	35 (22, 36)	36 (24, 36)	0.20
Age, years, mean (SD)	83 (3)	84 (4)	<0.001
Female sex, *N* (%)	101 (48)	279 (54)	0.13
Hypertension, *N* (%)	156 (74)	430 (83)	0.004
Heart failure, *N* (%)	87 (41)	272 (52)	0.005
Diabetes mellitus, *N* (%)	40 (19)	106 (20)	0.63
History of a thromboembolism, *N* (%)	16 (8)	122 (24)	<0.001
History of coronary artery disease, *N* (%)	36 (17)	88 (15)	0.60
CHADS_2_ score, mean (SD)	2.5 (1.0)	2.8 (1.1)	<0.001
CHA_2_DS_2_-VASc score, mean (SD)	4.2 (1.3)	4.3 (1.2)	0.09
Chronic kidney disease, *n* (%)	140 (66)	349 (67)	0.75
Creatinine clearance, mL/min, mean (SD)	50 (17)	46 (16)	<0.001
Paroxysmal AF, *n* (%)	134 (63)	292 (56)	0.08
Medication			
ACE-I/ARBs, *n* (%)	113 (53)	263 (51)	0.52
β-blockers, *n* (%)	118 (56)	251 (48)	0.07
DOACs, *n* (%)	212 (100)	570 (100)	1.0
Adequate DOAC dose, *n* (%)	177 (85)	471 (83)	0.78
Antiarrhythmic drugs, *n* (%)	36 (17)	57 (10)	0.007
TTE parameters			
LA diameter, mm, mean (SD)	38 (6)	40 (7)	0.006
LVEF, %, mean (SD)	63 (9)	60 (11)	<0.001

Values are presented as the mean ± SD or median (IQR) in accordance with the distribution and number of patients (%). Abbreviations: ACE-I: angiotensin-converting enzyme inhibitor; AF: atrial fibrillation; ARB: angiotensin II receptor blocker; DOAC: direct oral anticoagulant; IQR: interquartile range; LA: left atrium; SD: standard deviation; LVEF: left ventricular ejection fraction; TTE: transthoracic echocardiogram.

After propensity score matching, 208 patients were extracted from each group (Figure 1). Except for the LA diameter and LVEF, the baseline characteristics were well matched between the 2 groups (Table 2).

**Table 2:** Baseline characteristics (propensity score matched cohort).

	**Ablation group (*N* = 208)**	**Medication group (*N* = 208)**	***P*-value**
Follow-up period, months, median (IQR)	35 (22, 36)	36 (23, 36)	0.13
Age, years, mean (SD)	83 (3)	83 (3)	0.83
Female sex, *N* (%)	101 (49)	95 (46)	0.56
Hypertension, *N* (%)	152 (73)	152 (73)	1.0
Heart failure, *N* (%)	87 (42)	88 (42)	0.92
Diabetes mellitus, *N* (%)	40 (19)	42 (20)	0.81
History of a thromboembolism, *N* (%)	16 (8)	18 (9)	0.72
History of coronary artery disease, *N* (%)	36 (17)	44 (21)	0.32
CHADS_2_ score, mean (SD)	2.5 (1.0)	2.5 (1.0)	0.85
CHA_2_DS_2_-VASc score, mean (SD)	4.2 (1.3)	4.3 (1.3)	0.26
Chronic kidney disease, *n* (%)	137 (66)	137 (66)	1.0
Creatinine clearance, mL/min, mean (SD)	50 (17)	48 (18)	0.13
Paroxysmal AF, *n* (%)	130 (63)	131 (63)	0.92
Medication			
ACE-I/ARBs, *n* (%)	109 (52)	100 (48)	0.38
β-blockers, *n* (%)	114 (55)	105 (50)	0.38
DOACs, *n* (%)	208 (100)	208 (100)	1.0
Adequate DOAC dose, *n* (%)	174 (84)	164 (79)	0.21
Anti-arrhythmic drugs, *n* (%)	36 (17)	24 (12)	0.09
TTE parameters			
LA diameter, mm, mean (SD)	38 (6)	40 (7)	0.034
LVEF, %, mean (SD)	63 (9)	61 (11)	0.005

Values are presented as the mean ± SD or median (IQR) in accordance with the distribution and number of patients (%). Abbreviations: ACE-I: angiotensin-converting enzyme inhibitor; AF: atrial fibrillation; ARB: angiotensin II receptor blocker; DOAC: direct oral anticoagulant; IQR: interquartile range; LA: left atrium; SD: standard deviation; LVEF: left ventricular ejection fraction; TTE: transthoracic echocardiogram.

### AF ablation

Among the 212 patients in the Ablation group, all first-time ablation procedures were successful. During the perioperative period, 9 (4.2%) complications occurred, in which 3 (1.4%) were cardiac tamponades, but all were managed either with treatment or observation. No fatal procedure-related complications occurred (Supplementary Table 1). After the initial ablation, 54 (24%) patients had an AF recurrence. Among them, 17 patients underwent a second ablation session. No complications occurred during the operative period. No patients underwent ≥3 ablation sessions. The incidence of an AF recurrence was similar regardless of the AF type (paroxysmal AF: *n* = 34/134 (25%) vs. non-paroxysmal AF: *n* = 20/78 (26%)). The patients with paroxysmal AF underwent a second ablation session more frequently than those with non-paroxysmal AF (*n* = 13/118 (9.7%) vs. *n* = 4/78 (5.1%)). The details of the AF characteristics and management including of an AF recurrence, specific procedures in the second ablation session, and anti-arrhythmic drug use in the Ablation group patients for each AF type are shown in Supplementary Table 2.

### Outcomes

**Figure 2:**
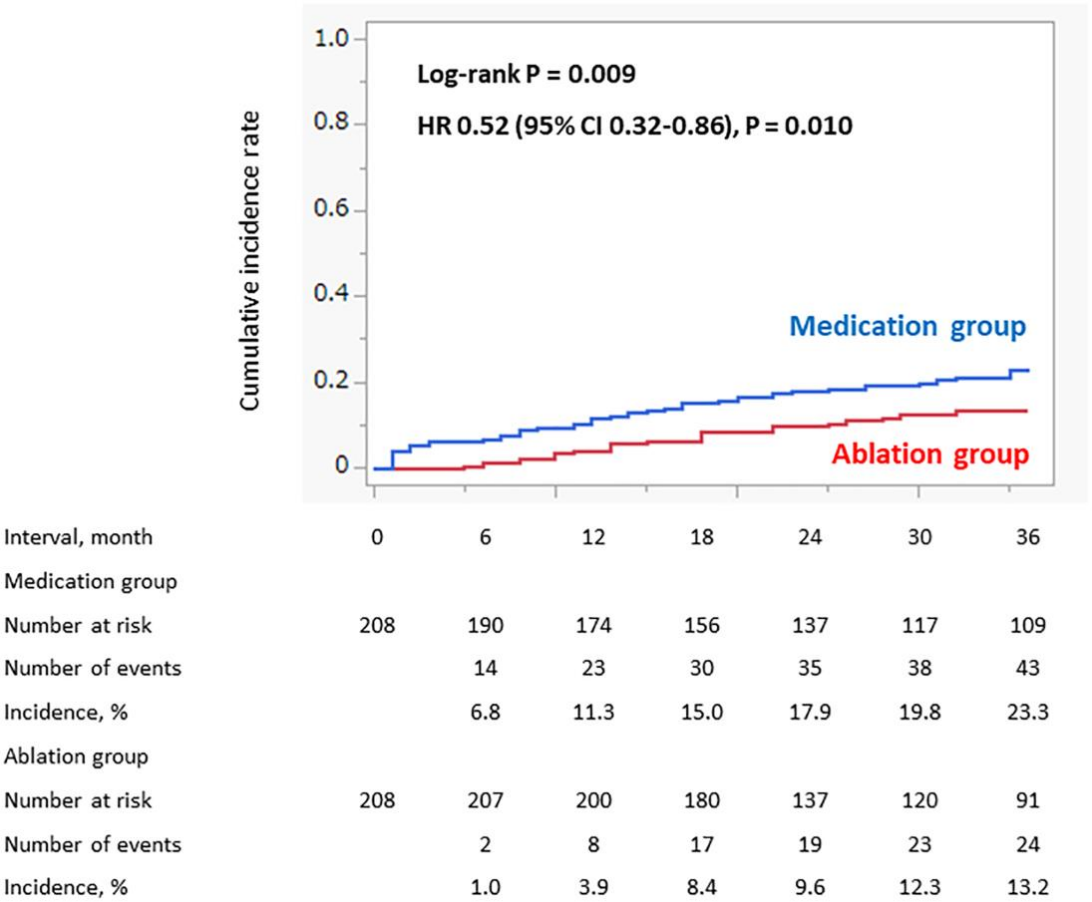
**Incidence of cardiovascular events (propensity score matched cohort).** The incidence of the cardiovascular events within 3 years was significantly lower in the Ablation group than in the Medication group.

As the primary outcome, the 3-year incidence of cardiovascular events was significantly lower in the Ablation group than in the Medication group: 24 (13.2%) vs. 43 (23.3%), log-rank *P* = 0.009 and hazard ratio (HR) 0.52 (95% confidence interval (CI) 0.32–0.86) (Figure 2). Among the cardiovascular events, only the incidence of acute heart failure (AHF) significantly differed (*P* = 0.021). The incidence of stroke and systemic embolism (SSE) was higher in the Ablation group than in the Medication group, however, there was no statistically difference (Table 3). Among the SSE, the incidence of hemorrhagic stroke was higher in the Ablation group than in the Medication group (*n* = 5 (2.4%) vs. *n* = 1 (0.5%)), and, the incidence of ischemic stroke was similar between the groups (*n* = 4 (1.9) vs. *n* = 5 (2.4%)).

**Table 3:** Details of the cardiovascular events and deaths (propensity score matched cohort).

	**Ablation group (*N* = 208)**	**Medication group (*N* = 208)**	**HR (95% CI)**	***P*-value**
Cardiovascular events, *N* (%)	24 (11.5)	43 (20.7)	0.52 (0.32–0.86)	0.010
Acute heart failure, *N* (%)	13 (6.3)	27 (13.0)	0.45 (0.24–0.89)	0.021
Stroke and systemic embolism, *N* (%)	9 (4.3)	5 (2.4)	1.76 (0.59–5.24)	0.31
Acute coronary syndrome, *N* (%)	1 (0.5)	5 (2.4)	0.20 (0.02–1.68)	0.14
Cardiovascular deaths, *N* (%)	5 (2.4)	15 (7.2)	0.32 (0.12–0.88)	0.027
Acute heart failure, *N* (%)	0 (0)	7 (3.4)	–	–
Stroke and systemic embolism, *N* (%)	1 (0.5)	1 (0.5)	0.96 (0.06–15.4)	0.98
Acute coronary syndrome, *N* (%)	0 (0)	1 (0.5)	–	–
Sudden cardiac death, *N* (%)	4 (1.9)	6 (2.9)	0.64 (0.18–2.28)	0.49

Values are presented as the number of patients (%). Abbreviation: HR: hazard ratio.

The percentage of patients with ≥2 cardiovascular events was lower in the Ablation group than in the Medication group (*n* = 3 (1.4%) vs. *n* = 8 (3.8%)).

**Figure 3:**
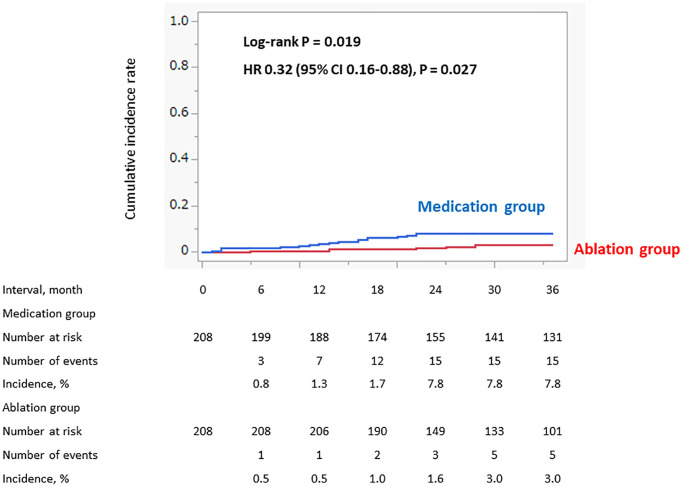
**Incidence of cardiovascular death (propensity score matched cohort).** The incidence of the cardiovascular death within 3 years was significantly lower in the Ablation group than the Medication group.

As the secondary outcome, the 3-year incidence of cardiovascular death was also significantly lower in the Ablation group than in the Medication group: 5 (3.0%) vs. 15 (7.8%), log-rank *P* = 0.019, HR 0.32 (95% CI 0.16–0.88) (Figure 3). No AHF/acute coronary syndrome (ACS)-related deaths occurred in the Ablation group. The incidence of a SSE-related death and sudden cardiac death (SCD) were similar between the groups (Table 3).

**Figure 4:**
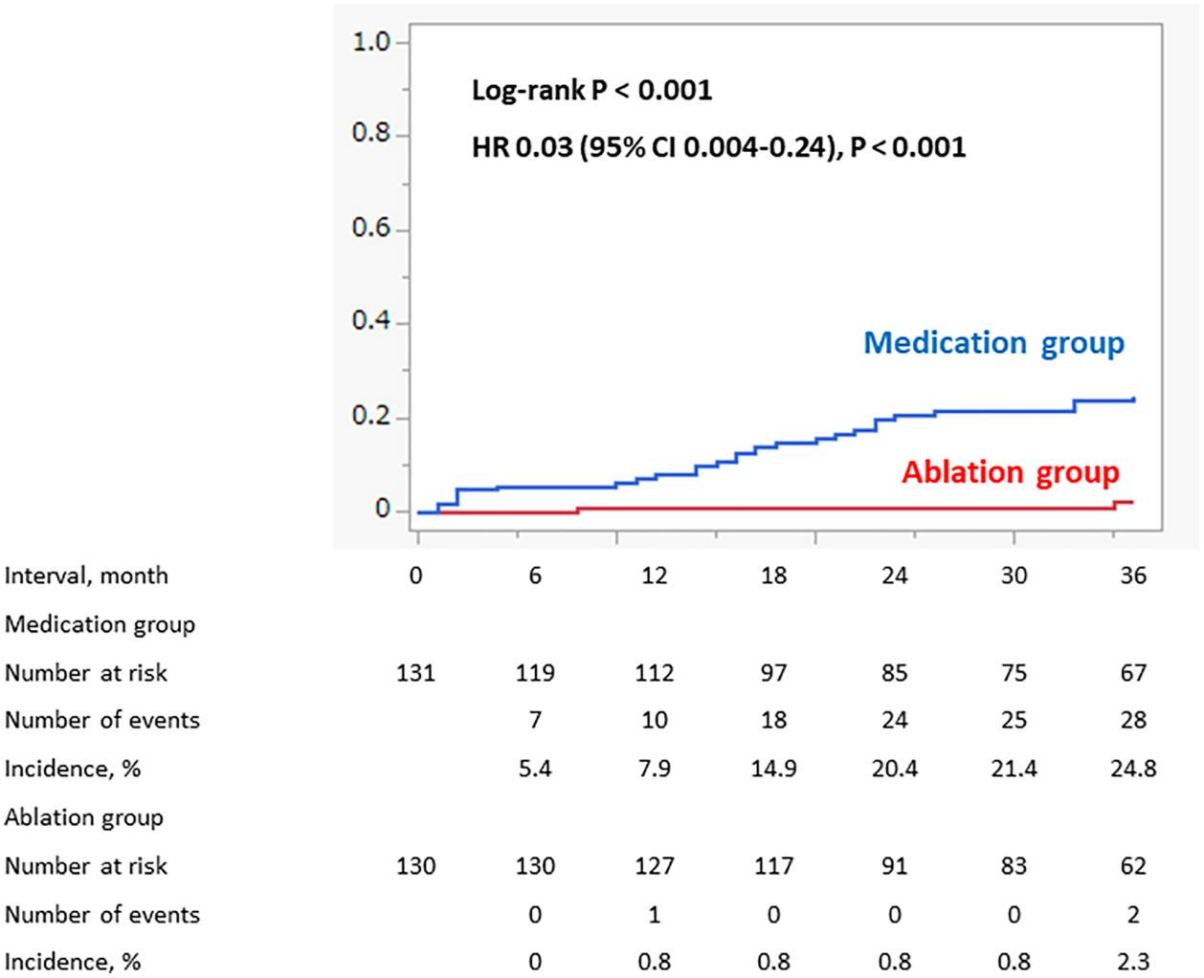
**Progression to permanent AF in patient with paroxysmal AF at baseline (propensity score matched cohort).** Among the patients with paroxysmal AF, the incidence of the progression from paroxysmal to permanent AF was significantly lower in the Ablation group than in the Medication group.

The percentage of patients with non-paroxysmal AF at baseline was similar between the Ablation and Medication groups (*n* = 78 (37.5%) vs. *n* = 77 (37%)). Although all patients in the Ablation group regained sinus rhythm after ablation, sinus rhythm was not maintained in 5 patients, and AF became a permanent form during the follow-up period. In contrast, 28 paroxysmal AF patients in the Medication group progressed to permanent AF through a natural course. As a result, the percentage of patients with permanent AF during the follow-up was significantly lower in the Ablation group than in the Medication group (*n* = 5 (2.4%) vs. *n* = 105 (50.4%), *P* < 0.001). Among the patients with paroxysmal AF, the incidence of the progression from paroxysmal to permanent AF was significantly lower in the Ablation group than in the Medication group: 2 (2.3%) vs. 28 (24.8%), log-rank *P* < 0.001, HR 0.03 (95% CI 0.004–0.24) (Figure 4). The number of patients that required pacemaker implantations during the follow-up period was similar between the Ablation group and the Medication group (*n* = 12 (5.8%) vs. 13 (6.3%)).

Sensitivity analyses of the primary and secondary outcomes in the original total cohort exhibited comparable results (Supplementary Figures 1 and 2). The incidence of a cardiovascular event and cardiovascular death were significantly and numerically lower, respectively, in the Ablation group than in the Medication group (Supplementary Table 3). After adjusting for the risk factors of cardiovascular events, the multivariate Cox proportional hazard model determined that catheter ablation was an independent predictor of a non-cardiovascular event occurrence (Supplementary Table 4). In terms of cardiovascular death, the HR of catheter ablation was 0.45, however, it was not statistically significant (Supplementary Table 5). In the original total cohort, the 3-year incidence of non-cardiovascular death was significantly higher in the Medication group than the Ablation group, however, that was similar between the two groups in the propensity score matched cohort (Supplementary Figures 3 and 4). The details of the non-cardiovascular deaths in the total original and propensity-matched cohorts are shown in Supplementary Tables 6 and 7.

## DISCUSSION

Our study indicated the preventive effects of catheter ablation on both cardiovascular events and death in AF patients aged ≥80 years using DOACs in the propensity score matched cohort. Those results were consistently found in the original total cohort, including the multivariate Cox proportional hazard model adjusting for the clinically relevant risk factors. The usage rate of DOACs in the very old patients who fulfilled the indications for DOACs was 54.3%. Of those using DOACs, AF ablation was performed in 27.1%. The complication rate of AF ablation was 4.2%, and no fatal events occurred. Of those who underwent AF ablation, permanent AF recurred in 2.4% during the follow-up period.

The EAST-AFNET 4 trial reported that early rhythm control therapy with antiarrhythmic drugs or AF ablation was associated with a lower risk of adverse cardiovascular outcomes than usual care among patients with AF and those of all age subgroups including those aged ≥75 years [12]. However, the effect of AF ablation as a powerful rhythm control therapy on the hard endpoints, including ischemic strokes and mortality, remains controversial even among the younger population as shown in the CABANA trial [6]. Furthermore, evidence among the older people is limited. Although recent studies, including a meta-analysis, have shown the effect of AF ablation among older people, most studies reported the procedural outcomes and/or AF recurrence [13, 14]. There were a few studies that mentioned the beneficial effects on the long-term hard endpoints among older people. An observational study that included patients aged ≥75 years demonstrated that cardiovascular mortality was significantly lower in 571 ablated patients than in 571 non-ablated patients in the propensity score matched cohort during a mean follow-up period of 39.75 months [15]. However, the incidence of strokes/transient ischemic attacks and cardiovascular hospitalizations did not differ between the 2 groups. In that study, ≥90% of the patients received anticoagulation therapy, and numerous cardiovascular hospitalizations other than AF-related events increased in the older AF patients, which were the reasons considered to have contributed to the results. Our cardiovascular events were strictly defined as AF-related events. As a result, the incidence of cardiovascular events was significantly lower in the patients who underwent AF ablation. Moreover, the patients included in this study were aged ≥80 years old, and therefore, had a higher risk of cardiovascular events, which could have led to those results.

The main benefit of AF ablation is the prevention of AF progression. In paroxysmal AF, the frequency and duration could at least be reduced by ablation. In persistent AF, ablation may result in conversion to paroxysmal AF, meaning AF regression. Several studies have demonstrated that the incidence of cardiovascular events and mortality are higher in patients with non-paroxysmal AF than in those with paroxysmal AF [16, 17]. In addition, a recent study suggested that cardiovascular events frequently occur during the period wherein AF progresses from a paroxysmal to persistent type [18]. Therefore, the preventive effects of AF ablation on AF progression would contribute to a lower incidence of cardiovascular events and death. Procedural safety is a major concern in the older AF population that undergo AF ablation. Several studies have reported that the procedural complications following AF ablation are higher in older patients than younger patients [13, 19]. A recent nationwide database study containing >135,000 patients (aged 65 ± 10 years) showed that the overall in-hospital complication rate was 3.4% (cardiac tamponade: 1.2%) and that an increased age was independently associated with the overall complications; the adjusted odds ratio of 80–84 years was 1.90 and that of ≥85 years was 2.86 when the reference was <60 years [19]. In this study, the perioperative complication rate was 4.2% (cardiac tamponade: 1.4%) and was similar to that of the overall complication rate in the recent study, and therefore, the safety of our procedure in older patients could be acceptable. Furthermore, the procedural efficacy and safety of the latest ablation technologies, which have been reported in patients aged ≥80 years [20, 21], could support our results.

Careful patient selection is also an important issue. Based on our data, the characteristics in those whom ablation therapy is recommended would be considered from 3 perspectives: procedural efficacy and safety, risk of AF-related adverse events, and symptom severity. First, the procedural efficacy depends on the type of AF and LA size in general. Most Ablation group patients had paroxysmal or short duration persistent AF (median AF persistence duration: 4 months) and less than mild LA enlargement (mean LA diameter: 38 mm). The Medication group patients in the propensity score matched cohort had more paroxysmal AF than those in the original total cohort. Procedural safety may depend on the sex and comorbidities. A large-scale study also reported that independent predictors, other than a higher age, were a female sex and comorbidities [19]. The Medication group patients in the propensity score matched cohort had a lesser proportion of females and comorbidities, such as hypertension, heart failure, a history of a thromboembolism, and a reduced renal function, as compared to those in the original total cohort. Second, the risk of AF-related adverse events would be considered. AF ablation therapy is considered to be more effective for the prognosis in patients with a higher risk of AF-related events, especially worsening heart failure. From this perspective, patients with a higher age, females, non-paroxysmal AF, and more comorbidities might benefit from AF ablation in terms of the prognosis. Although this perspective may be inconsistency with that of procedural safety, advances in the technologies and operators’ experience could complement the procedural safety as shown in our study. In fact, the Ablation group patients had a relatively higher proportion of females (48%), non-paroxysmal AF (37%), hypertension (74%), heart failure (41%), and chronic kidney disease (66%). Ablation in such high-risk patients, especially those with a history of AF-induced heart failure, would be recommended even among the older population. Finally, symptom severity should be mentioned. Around 90% of the Ablation group patients had symptoms bad enough to hope for an invasive therapy. In general, patients with paroxysmal AF have a favorable indication for ablation therapy because of the high non-recurrence rate, and therefore, those with asymptomatic paroxysmal AF may be also considered. However, older patients have a higher recurrence rate even of paroxysmal AF due to the frequent occurrence of non-PV foci [14]. In fact, 25% of patients with paroxysmal AF had an AF recurrence in our study. Therefore, the symptom severity is basically considered especially among the older patients.

To the best of our knowledge, this is the first study to have demonstrated the preventive effect of AF ablation on long-term AF-related cardiovascular events in very old patients with NVAF. Our findings would add new insight into AF management to improve the prognosis among the older population. Only treatment with medications is insufficient even in the DOAC era, and a rhythm control strategy by catheter ablation is effective and should be encouraged in selected patients. Our results could contribute to real-world clinical practice under circumstances with an increase in the AF population along with a worldwide aging society.

Several limitations should be considered. First, this was a single-center observational study, and we could have investigated the detailed clinical courses and outcomes. Second, selection bias was inevitable because this study was conducted in a high-volume center where ≥400 AF ablation procedures were performed per year. However, our study provided advanced real-world data in very old AF patients because experienced physicians and operators decided the appropriate medical treatment for each patient. Third, the preventive effects of AF ablation on SSEs remained unclear due to the similar or higher incidence of SSEs in the Ablation group than the Medication group. Although those findings might suggest the impact of DOACs on SSE prevention, further study is required to clarify the effect of AF ablation on SSE prevention among the older population. Finally, this study was non-randomized, and the size of the effect would have been biased with other unknown confounders, even though we used the propensity score matching method. In addition, the patients’ frailty and drug adherence could not be evaluated despite being key factors in older patients. Therefore, there might have been some question about the patient selection; relatively fewer sick older patients were considered for an indication of catheter ablation. However, we tried to adjust that as much as possible with a multivariate Cox proportional hazard model including the body weight in the original total cohort, and the effects of the catheter ablation were consistently found. In addition, the similar incidence of non-cardiovascular death in the propensity score matched cohort could support the main findings of the study. A larger multicenter randomized study is needed to validate the generalization of our results, however, it would be difficult to perform that in older people.

## CONCLUSIONS

NVAF patients aged ≥80 years who underwent AF ablation and used DOACs had a lower incidence of cardiovascular events and death as compared to those who received medical therapy alone. AF ablation could be a preferable treatment option to improve the prognosis in selected older patients, even in the era of DOACs.

## METHODS

### Study design and patients

We conducted a prospective cohort study of consecutive patients with AF aged ≥80 years who received medical treatment from March 2011 to August 2021 at Kagawa Prefectural Central Hospital, Kagawa, Japan. The inclusion criteria were as follows: 1) diagnosed with AF; 2) age ≥80 years at the time of enrollment; and 3) DOAC use at the time of enrollment. The exclusion criteria were as follows: 1) presence of valvular AF; 2) end-stage renal failure (creatinine clearance <15 mL/min/1.73 m^2^ or regular dialysis); and 3) underwent AF ablation before 80 years of age. Valvular AF was defined as moderate or severe mitral stenosis or having undergone a mechanical valve replacement.

We basically decided the indication of AF ablation for patients with symptomatic AF after confirming their preserved cognitive function and a self-supporting ability for performing daily life. We also considered those who were expected to have a high procedural efficacy and safety, such as lesser LA enlargement and comorbidities. For patients with non-paroxysmal AF, we basically considered those with an AF persistence duration of less than 12 months and in their early 80’s as indicated. For patients with asymptomatic AF, we proposed ablation therapy as a treatment option especially for those who had a risk of AF-related events, such as a history of heart failure, if they were expected to have a high procedural efficacy and safety. Aside from those, AF ablation was performed based on the decision of the patients, their families, and their attending physicians.

This study conformed to the principles of the Declaration of Helsinki and was conducted after approval from the Clinical Ethics Committee of the Kagawa Prefectural Central Hospital. Written informed consent was substituted by an opt-out method by announcing the handling of the personal data and right to withdraw consent on the website of the study institution.

### AF ablation

DOACs were initiated at least a month before the ablation and continued even after the ablation unless DOAC-related adverse events occurred. Transesophageal echocardiography was performed to confirm the absence of any LA thrombi on the day of admission for the ablation.

AF ablation was performed based on the established method [11]. Briefly, electrical pulmonary vein isolation by radiofrequency ablation was performed using an open-irrigation 3.5-mm tip deflectable catheter (Thermocool; Biosense Webster, CA, USA) with contact-force sensing and a 3-dimensional mapping system (CARTO; Biosense, Webster, CA, USA) or cryoablation using a second generation cryoballoon (Arctic Front Advance; Medtronic, MN, USA). We used heparin to maintain the activated clotting time at ≥300 seconds during the procedure. Regarding the radiofrequency ablation, we conducted a traditional ablation strategy delivering radiofrequency energy at a power output of 25–40 W and with a maximum temperature of 45°C. We performed a force-time integral guided ablation until May 2018 and then an ablation index guided ablation from June 2018 to July 2021. The target value of the force-time integral was 80 gram-seconds with a power output delivery of 35 W on the anterior wall, 30 W on the posterior wall, and 25 W in front of the esophagus using a dragging ablation technique. The target value of the ablation index was 350–400 with 35–40 W on the anterior wall, 300–350 with 30–35 W on the posterior wall, and 250 with 30–35 W in front of the esophagus. We set the ablation index values slightly lower considering the safety. Instead, we set the minimum lesion distance closer (≤3 mm). A posterior wall isolation, superior vena cava isolation, and cavotricuspid isthmus ablation were performed as needed. General anesthesia was induced and maintained with a continuous propofol intravenous infusion using a subglottic device to keep the airway open and an artificial respirator to maintain effective ventilation. We tried to avoid multiple ablation sessions by using low-dose antiarrhythmic drugs when AF recurred. We performed a second ablation session depending on their symptom severity: uncontrolled severe symptoms and frequent occurrence while using drugs. During the second ablation session, we checked for pulmonary vein reconnections of the block lines created during the first ablation session and performed a re-isolation if reconnections were identified. A posterior wall isolation, low-voltage area or trigger site ablation, and linear ablation, including a roof line and mitral isthmus ablation, were performed as needed.

After the ablation, 12-lead ECGs were performed every 3 months at clinical visits. Furthermore, 12-lead ECGs or 24-h Holter ECGs were performed as needed, such as at the time of the symptom occurrence. AF recurrence was defined as an atrial tachyarrhythmia, including AF and atrial tachycardia, which was detected on a 12-lead ECG, or an atrial tachyarrhythmia lasting at least 30 seconds that was documented on a 24-h Holter ECG. A permanent form of AF was defined as an atrial tachyarrhythmia occurring all the time, and more than 3 times, with ECGs, which were evaluated at more than one-month intervals.

### Data collection and outcomes

We collected data on the patient characteristics, including the medical history, laboratory data, medication use, and electrocardiography and TTE findings. TTE was performed to measure the LA diameter and LVEF. The LVEF was measured using the disk summation method and expressed as the average over five cardiac cycles if AF persisted. We investigated the outcomes from the medical records at Kagawa Prefectural Central Hospital. We further investigated the outcomes in March 2021 by a mail-in questionnaire or telephone call to the patients, families, and their primary care physicians in the patients who completed clinical visits at Kagawa Prefectural Central Hospital.

The primary outcome was an initial cardiovascular event, and the secondary outcome was cardiovascular death. We defined cardiovascular events as AHF requiring hospitalizations, SSEs, ACS, and SCD. We defined cardiovascular death as death caused by AHF, SSEs, ACS, and SCD.

### Statistical analysis

We compared the patient background and outcomes between the Ablation and Medication groups. The primary analysis of this study was in the propensity score matched cohort followed by sensitivity analyses in the original total cohort. We used the age, sex, creatinine clearance, prevalence of hypertension, heart failure, and diabetes mellitus, and history of thromboembolisms and coronary artery disease in the logistic regression model to develop the propensity score for the ablation use. We then constructed a 1:1 matched pair for the Ablation and Medication groups with a tolerance of 0.03 based on the propensity score.

Continuous variables were expressed as the mean and standard deviation or median and interquartile range for their distribution. We used the *t*-test or Wilcoxon rank-sum test for the between-group comparisons. Categorical variables are presented as numbers and percentages, and the intergroup comparisons were conducted using the Chi-square test. The outcomes are presented as the number of initial events. The Kaplan–Meier survival curve and log-rank test were used to compare the incidence rates of the outcomes of the Ablation and Medication groups considering the censoring due to the end of follow-up without outcomes. The effect was expressed as the HR and its 95% CI with the use of a Cox proportional hazard model.

In the propensity score matched cohort, we compared the percentage of the patients with non-paroxysmal AF at baseline and during the follow-up in each group. Among them, we compared the incidence of the progression from a paroxysmal to permanent form during the follow-up in patients with paroxysmal AF.

We also performed a sensitivity analysis for the primary and secondary outcomes on the original total cohort in the same way as the main analysis. A multivariable Cox proportional hazard model was used to determine the factors associated with the primary and secondary outcomes for the purpose of minimizing the selection bias. The clinically relevant candidate variables for cardiovascular events included catheter ablation, an age ≥85 years, female sex, body weight ≤50 kg, estimated glomerular filtration rate <60 mL/min/1.73 m^2^, heart failure, hypertension, diabetes mellitus, history of a thromboembolism, and non-paroxysmal AF. The candidate variables for cardiovascular death included catheter ablation, and an age ≥85 years, body weight ≤50 kg, heart failure, and non-paroxysmal AF. We also investigated the 3-year incidence of non-cardiovascular death as a sensitivity analysis in the propensity score matched and original total cohorts to confirm the accuracy of the propensity score matching.

Two-sided *P*-values < 0.05 were considered statistically significant. All statistical analyses were performed using JMP version 15 software (SAS Institute Inc., Cary, NC, USA) and SPSS Statistics version 24 software (IBM Institute Inc. Chicago, IL, USA).

## Supplementary Materials

Supplementary Figures

Supplementary Tables
