# Supplementary material for: Cardiovascular events and death after catheter ablation in very old patients with nonvalvular atrial fibrillation
Source: Aging (Albany NY). 2023 Aug 14;15(15):7343–61. doi: 10.18632/aging.204952 (PMC10457051; doi:10.18632/aging.204952)
Supplement: Supplementary Figures [file aging-15-204952-s001.pdf]

SUPPLEMENTARY FIGURES

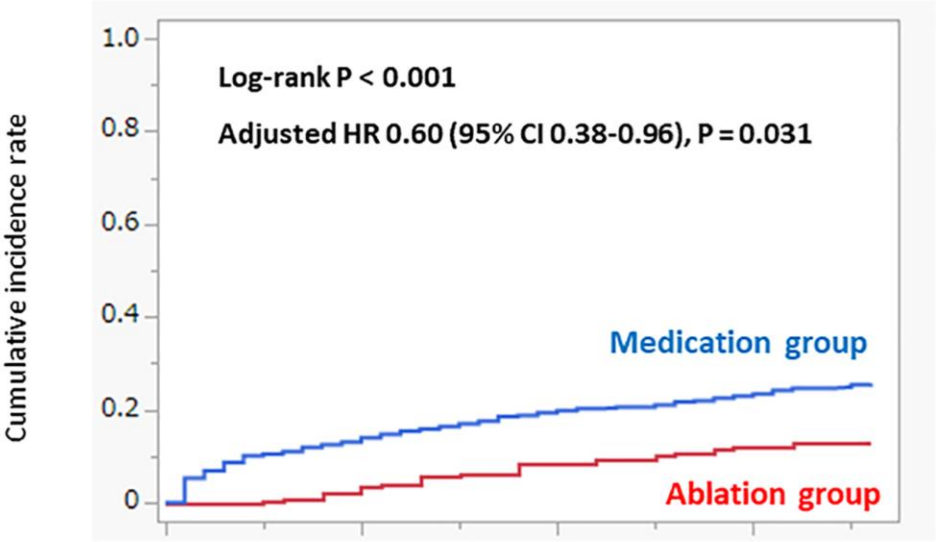

Interval, month	0	6	12	18	24	30	36
Medication group							
Number at risk	570	497	447	408	367	318	280
Number of events		62	86	103	112	124	133
Incidence, %		11.0	15.5	18.8	20.6	23.4	25.1
Ablation group							
Number at risk	212	211	204	184	139	121	91
Number of events		2	8	17	19	23	24
Incidence, %		0.9	3.8	8.2	9.5	12.2	12.9

Supplementary Figure 1. Incidence of cardiovascular events (original total cohort). The incidence of cardiovascular events within 3 years was significantly lower in the Ablation group than Medication group.

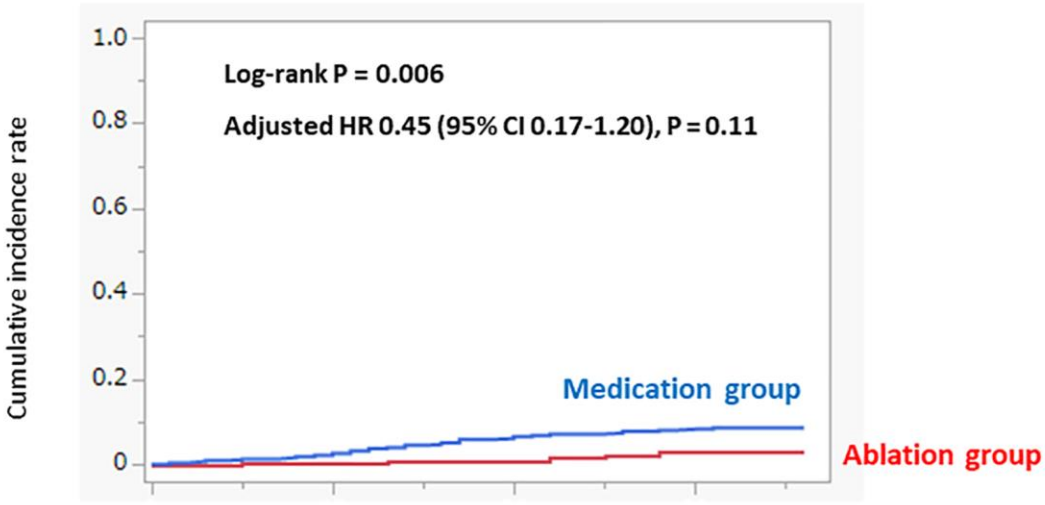

Interval, month	0	6	12	18	24	30	36
Medication group							
Number at risk	570	542	502	468	428	375	339
Number of events		7	20	31	37	42	43
Incidence, %		1.3	3.7	5.9	7.1	8.3	8.5
Ablation group							
Number at risk	212	212	210	194	151	134	101
Number of events		1	1	2	3	5	5
Incidence, %		0.5	0.5	1.0	1.6	2.9	2.9

Supplementary Figure 2. Incidence of cardiovascular death (original total cohort). The incidence of cardiovascular death within 3 years was significantly lower in the Ablation group than Medication group.

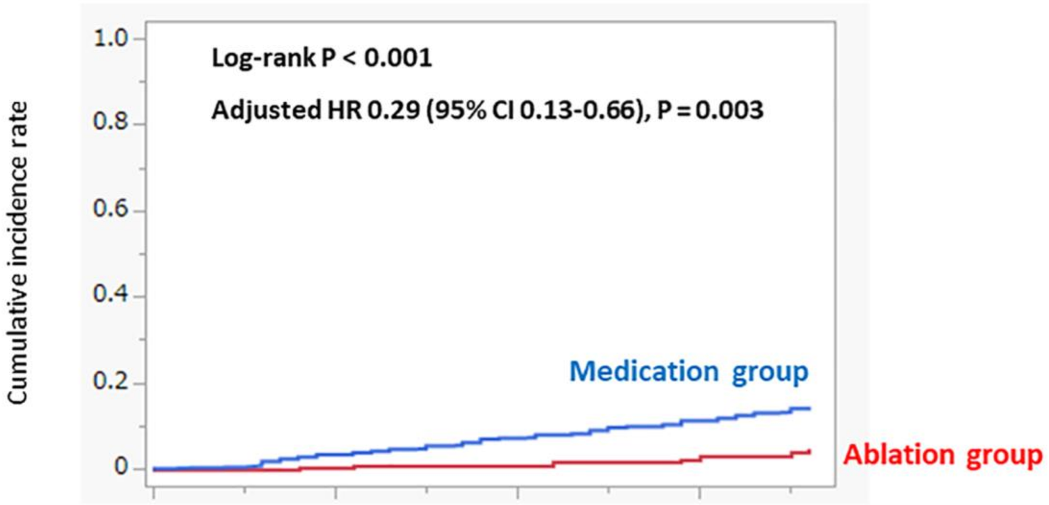

Interval, month	0	6	12	18	24	30	36
Medication group							
Number at risk	570	542	502	468	428	375	339
Number of events		9	22	36	46	56	69
Incidence, %		1.6	4.1	6.8	8.9	11.2	14.4
Ablation group							
Number at risk	212	212	210	194	151	134	101
Number of events		0	2	2	3	5	7
Incidence, %		0	1.0	1.0	1.6	3.0	4.9

Supplementary Figure 3. Incidence of non-cardiovascular death (original total cohort). The incidence of non-cardiovascular death within 3 years was significantly lower in the Ablation group than Medication group.

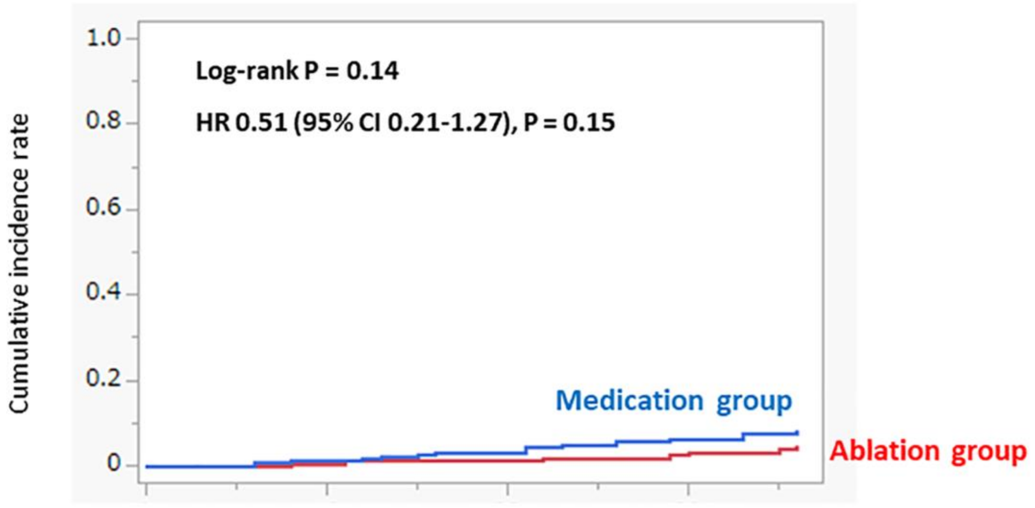

Interval, month	0	6	12	18	24	30	36
Medication group							
Number at risk	208	199	188	174	155	141	131
Number of events		1	3	6	9	11	14
Incidence, %		0.5	1.5	3.2	4.9	6.2	8.3
Ablation group							
Number at risk	208	208	206	190	149	133	101
Number of events		0	2	2	3	5	7
Incidence, %		0	1.0	1.0	1.6	3.0	4.9

Supplementary Figure 4. Incidence of non-cardiovascular death (propensity score matched cohort). The incidence of non-cardiovascular death within 3 years was similar between the Ablation and Medication groups.
