# Supplementary material for: Cardiovascular events and death after catheter ablation in very old patients with nonvalvular atrial fibrillation
Source: Aging (Albany NY). 2023 Aug 14;15(15):7343–61. doi: 10.18632/aging.204952 (PMC10457051; doi:10.18632/aging.204952)
Supplement: Supplementary Tables [file aging-15-204952-s002.pdf]

SUPPLEMENTARY TABLES

Supplementary Table 1. Details of the complications during the first-time ablation procedure.

	Ablation group (*N* = 212)
Perioperative complications, *N* (%)	9 (4.2)
Cardiac tamponade, *N* (%)	3 (1.4)
Sinus node injury, *N* (%)	1 (0.5)
Periesophageal vagal nerve injury, *N* (%)	1 (0.5)
Phrenic nerve injury, *N* (%)	3 (1.4)
Pseudo aneurysm on puncture site, *N* (%)	1 (0.5)
Death, *N* (%)	0 (0)

Values are presented as the number of patients (%).

Supplementary Table 2. Details of the AF characteristics and management in the ablation group patients.

	Patients with paroxysmal AF (*N* = 134)	Patients with non-paroxysmal AF (*N* = 78)
Symptomatic AF, *N* (%)	118 (88)	63 (81)
AF persistence duration, month, median (IQR)	–	4 (2, 6)
Long-standing persistent AF, *N* (%)	–	3 (3.8)
AF recurrence after first-time ablation, *N* (%)	34 (25)	20 (26)
Second-time ablation, *N* (%)	13 (9.7)	4 (5.1)
Redo PV isolation, *N*	5	3
Redo SVC isolation, *N*	1	1
SVC isolation, *N*	5	0
Posterior wall isolation, *N*	3	2
Mitral isthmus ablation, *N*	2	1
Roofline ablation, *N*	2	0
Trigger ablation, *N*	2	0
Low voltage zone ablation, *N*	2	0
Complication, *N*	0	0
Permanent form of AF fixing, *N*, (%)	2 (1.5)	3 (3.8)
Anti-arrhythmic drug use, *N* (%)	17 (13)	18 (23)
Amiodarone 50 mg/day, *N*	6	5
Amiodarone 100 mg/day, *N*	1	2
Amiodarone 200 mg/day, *N*	0	1
Bepridil 25 mg/day, *N*	0	3
Bepridil 50 mg/day, *N*	7	6
Bepridile 100 mg/day, *N*	0	1
Pilsicainide 50 mg/day, *N*	1	0
Pilsicainide 100 mg/day, *N*	1	0
Cibenzoline 50 mg/day, *N*	1	0
Aprindine 40 mg/day, *N*	2	0

Values are presented as the number of patients (%).

Supplementary Table 3. Details of the cardiovascular events and deaths (original total cohort).

	Ablation group (*N* = 212)	Medication group (*N* = 570)	Adjusted HR (95% CI)	*P*-value
Cardiovascular events, *N* (%)	24 (9.4)	133 (23.3)	0.60 (0.38–0.96)	0.031
Acute heart failure, *N* (%)	13 (6.1)	79 (13.9)	0.53 (0.28–0.98)	0.045
Stroke and systemic embolism, *N* (%)	9 (4.2)	38 (6.7)	0.93 (0.42–2.02)	0.85
Acute coronary syndrome, *N* (%)	1 (0.5)	8 (1.4)	0.29 (0.03–2.63)	0.27
Cardiovascular deaths, *N* (%)	5 (2.4)	43 (7.4)	0.45 (0.17–1.20)	0.11
Acute heart failure, *N* (%)	0 (0)	16 (2.8)	–	–
Stroke and systemic embolism, *N* (%)	1 (0.5)	10 (1.8)	0.47 (0.05–4.05)	0.49
Acute coronary syndrome, *N* (%)	0 (0)	2 (0.3)	–	–
Sudden cardiac death, *N* (%)	4 (1.9)	15 (2.6)	0.94 (0.29–3.09)	0.92

Values are presented as the number of patients (%). Abbreviation: HR: hazard ratio.

Supplementary Table 4. Predictors of cardiovascular events (original total cohort).

	Univariable			Multivariable		
	HR	95% CI	*P*-value	HR	95% CI	*P*-value
Heart failure	2.77	1.98–3.88	<0.001	2.49	1.77–3.52	<0.001
Non-paroxysmal AF	1.71	1.20–2.44	0.003	1.62	1.11–2.36	0.012
Age ≥85 years	1.75	1.28–2.39	<0.001	1.46	1.05–2.02	0.024
History of a thromboembolism	1.47	1.01–2.13	0.042	1.47	1.00–2.16	0.048
Body weight ≤50 kg	1.59	1.16–2.18	0.004	1.32	0.94–1.86	0.11
Female sex	1.27	0.93–1.74	0.13	1.16	0.83–1.63	0.39
Diabetes mellitus	1.21	0.83–1.77	0.32	1.14	0.77–1.69	0.50
Hypertension	1.05	0.73–1.51	0.81	1.10	0.76–1.60	0.61
eGFR <60 mL/min/1.73 m^2	1.33	0.95–1.86	0.10	1.08	0.77–1.53	0.65
Catheter ablation	0.44	0.28–0.68	<0.001	0.60	0.38–0.96	0.031

Abbreviations: AF: atrial fibrillation; CI: confidence interval; eGFR: estimated glomerular filtration rate; HR: hazard ratio.

Supplementary Table 5. Predictors of cardiovascular death (original total cohort).

	Univariable			Multivariable		
	HR	95% CI	*P*-value	HR	95% CI	*P*-value
Heart failure	2.94	1.58–5.47	<0.001	2.46	1.31–4.61	0.005
Age ≥85 years	2.35	1.33–4.15	0.003	1.82	1.02–3.28	0.044
Body weight ≤50 kg	2.28	1.29–4.01	0.004	1.70	1.04–3.30	0.038
Non-paroxysmal AF	2.05	1.43–4.01	0.037	1.77	0.88–3.55	0.11
Catheter ablation	0.30	0.12–0.74	0.010	0.45	0.17–1.20	0.11

Abbreviations: AF: atrial fibrillation; CI: confidence interval; HR: hazard ratio.

Supplementary Table 6. Details of the non-cardiovascular deaths (original total cohort).

	Ablation group (*N* = 212)	Medication group (*N* = 570)	Adjusted HR (95% CI)	*P*-value
Non-cardiovascular deaths, *N* (%)	7 (3.3)	69 (12.1)	0.29 (0.13–0.66)	0.003
Infection, *N* (%)	0 (0)	15 (2.6)	–	–
Cancer, *N* (%)	3 (1.4)	13 (2.3)	0.51 (0.14–1.94)	0.32
Respiratory failure, *N* (%)	2 (0.9)	14 (2.5)	0.51 (0.11–2.43)	0.40
Other/unknown, *N* (%)	2 (0.9)	27 (4.7)	0.24 (0.05-1.03)	0.06

Values are presented as the number of patients (%). Abbreviation: HR: hazard ratio.

Supplementary Table 7. Details of the non-cardiovascular deaths (propensity score matched cohort).

	Ablation group (*N* = 208)	Medication group (*N* = 208)	HR (95% CI)	*P*-value
Non-cardiovascular deaths, *N* (%)	7 (3.4)	14 (6.7)	0.51 (0.21–1.27)	0.15
Infection, *N* (%)	0 (0)	3 (1.4)	–	–
Cancer, *N* (%)	3 (1.4)	5 (2.4)	0.65 (0.15–2.73)	0.56
Respiratory failure, *N* (%)	2 (1.0)	3 (1.4)	0.69 (0.11–4.10)	0.68
Other/unknown, *N* (%)	2 (1.0)	3 (1.4)	0.67 (0.11–4.01)	0.66

Values are presented as the number of patients (%). Abbreviation: HR: hazard ratio.
